# Challenges of Assessing Exon 53 Skipping of the Human *DMD* Transcript with Locked Nucleic Acid-Modified Antisense Oligonucleotides in a Mouse Model for Duchenne Muscular Dystrophy

**DOI:** 10.1089/nat.2023.0038

**Published:** 2023-11-24

**Authors:** Sarah Engelbeen, Daniel O'Reilly, Davy Van De Vijver, Ingrid Verhaart, Maaike van Putten, Vignesh Hariharan, Matthew Hassler, Anastasia Khvorova, Masad J. Damha, Annemieke Aartsma-Rus

**Affiliations:** ^1^ Department of Human Genetics, Leiden University Medical Center, Leiden, The Netherlands.; ^2^ University of Massachusetts Chan Medical School, RNA Therapeutics Institute, Worcester, Massachusetts, USA.; ^3^ Department of Chemistry, McGill University, Montreal, Canada.

**Keywords:** efficacy, locked nucleic acid, LNA, preclinical studies, exon skipping

## Abstract

Antisense oligonucleotide (AON)-mediated exon skipping is a promising therapeutic approach for Duchenne muscular dystrophy (DMD) patients to restore dystrophin expression by reframing the disrupted open reading frame of the *DMD* transcript. However, the treatment efficacy of the already conditionally approved AONs remains low. Aiming to optimize AON efficiency, we assessed exon 53 skipping of the *DMD* transcript with different chemically modified AONs, all with a phosphorothioate backbone: 2′-O-methyl (2′OMe), locked nucleic acid (LNA)-2′OMe, 2′-fluoro (FRNA), LNA-FRNA, αLNA-FRNA, and FANA-LNA-FRNA. Efficient exon 53 skipping was observed with the FRNA, LNA-FRNA, and LNA-2′OMe AONs in human control myoblast cultures. Weekly subcutaneous injections (50 mg/kg AON) for a duration of 6 weeks were well tolerated by hDMDdel52/*mdx* males. Treatment with the LNA-FRNA and LNA-2′OMe AONs resulted in pronounced exon 53 skip levels in skeletal muscles and heart up to 90%, but no dystrophin restoration was observed. This discrepancy was mainly ascribed to the strong binding nature of LNA modifications to RNA, thereby interfering with the amplification of the unskipped product resulting in artificial overamplification of the exon 53 skip product. Our study highlights that treatment effect on RNA and protein level should both be considered when assessing AON efficiency.

## Introduction

Duchenne muscular dystrophy (DMD) is a severe X-linked neuromuscular disorder affecting ∼1 in 5,000 newborn males and is caused by mutations in the *DMD* gene encoding for the protein dystrophin [1–3]. Dystrophin is an essential component of the dystrophin-glycoprotein complex which provides stability to muscle fibers during contractions. Due to frameshift or nonsense mutations in the *DMD* gene, no functional dystrophin is produced in DMD patients, which makes their muscles prone to contraction-induced damage [4]. Consequently, this leads to continuous cycles of degeneration and regeneration of muscle fibers until their regeneration capacity becomes depleted and fibers are replaced by fibrotic and adipose tissue. The loss of muscle tissue eventually leads to loss of ambulation and a need for assisted ventilation during the second decade of life. DMD patients eventually die in the third to fourth decade of life due to cardiorespiratory failure [1,2].

Becker muscular dystrophy is a milder and less progressive muscular dystrophy wherein mutations in the *DMD* gene maintain the reading frame giving rise to an internally deleted and partially functional dystrophin protein [4]. One promising approach to restore dystrophin production in DMD patients is by using antisense oligonucleotides (AONs) to skip out-of-frame exons to restore the reading frame. AONs are short, synthetic, single-stranded strands of nucleotides that bind to a target exon in the pre-mRNA dystrophin transcript, thereby hiding it from the splicing machinery which leads to the production of an in-frame dystrophin transcript, that can be translated into a partially functional, Becker-like, dystrophin protein [5,6].

AON-mediated exon skipping is a mutation specific approach. The largest group (∼28%) of DMD patients could benefit from AONs targeting exon 51 (13%), exon 45 (8.1%), or exon 53 (9.9%) [7]. Currently four exon skip AONs are conditionally approved for the treatment of DMD by the Food and Drug Administration; casimersen for exon 45, eteplirsen for exon 51 and viltolarsen and golodirsen for exon 53 [8–12], and one AON (viltolarsen) by the Japanese Ministry of Health, Labor, and Welfare [13]. Despite their conditional approval, restoration of only very low levels of dystrophin have been obtained till date. This is likely caused by insufficient delivery of AONs to muscles, as well as low exon-skipping efficacy [6].

One method to improve AON efficiency is through chemical modifications of the backbone and sugars. Backbone modifications include the phosphorothioate (PS) backbone, in which an oxygen of the phosphodiester bond is replaced by a sulfur atom, which increases the nuclease resistance but reduces the affinity for target RNA [14]. Sugar modifications where the 2′OH position of the ribose is substituted with 2′-O-methyl (2′OMe) or 2′-fluoro (FRNA) or where the ribose conformation is constrained by a methylene bridge between the 2′O and 4′ position (locked nucleic acid, LNA) further improve binding affinity and nuclear resistance [15,16].

The most commonly used mouse model for DMD, the *mdx* mouse, has a spontaneous nonsense mutation in exon 23 of the murine *Dmd* gene, and dystrophin expression can be restored by exon skipping. While AON stability and efficiency of mouse-specific AONs can be assessed in the *mdx* mouse, human-specific AONs cannot be assessed. Therefore, the hDMDdel52/*mdx* mouse, carrying the human *DMD* gene with a partial deletion of exon 52 on an *mdx* background, was created. Consequently, these mice lack dystrophin expression of both human and mouse origin. This model uniquely allows to test the effects of dystrophin restoration upon treatment with human specific AONs targeting exon 51 or exon 53 on muscle pathology and function [17,18].

In this study, we assessed treatment efficacy of different chemically modified human-specific AONs targeting exon 53 in hDMDdel52/*mdx* mice. While we initially observed high exon 53 skipping levels after treatment with LNA-2′OMe- or LNA-FRNA-modified AONs, this was not accompanied by increments in dystrophin restoration. This discrepancy could be largely explained by the interference of LNA-modified AONs on the amplification of the hDMDdel52, unskipped, product resulting in artificial overamplification of the exon 53 skip polymerase chain reaction (PCR) product. Our study suggests that caution should be taken when assessing AON efficiency and that both the treatment effect on RNA and protein level should be considered.

## Materials and Methods

### Antisense oligonucleotides

We designed AONs targeting exon 53 of the human *DMD* transcript, all with a PS backbone. The AONs were either fully modified with one (2′OMe, FRNA), or a combination of two (LNA-2′OMe, LNA-FRNA, αLNA-FRNA), or three (FANA-LNA-FRNA1, FANA-LNA-FRNA2) sugar modifications (Table 1).

**Table 1.** Sequence and Melting Temperature of Antisense Oligonucleotides Targeting Exon 53 of the Human *DMD* Transcript

Modification	Sequence (5*′*-3*′*)	*T_m_* (°C)
FANA-LNA-FRNA1	(nC)^*^(nT)^*^(nG)^*^(nT)^*^(lT)^*^(fG)^*^(fC)^*^(lC)^*^(fU)^*^(fC)^*^(lC)^*^(nG)^*^(nG)^*^(nT)^*^(nT)^*^(nC)^*^(nT)^*^(nG)	>90
FANA-LNA-FRNA2	(nC)^*^(nT)^*^(nG)^*^(nT)^*^(lT)^*^(fG)^*^(nC)^*^(lC)^*^(fU)^*^(nC)^*^(lC)^*^(nG)^*^(nG)^*^(nT)^*^(nT)^*^(nC)^*^(nT)^*^(nG)	>85
αLNA-FRNA	(fC)^*^(fU)^*^(fG)^*^(fU)^*^(αlT)^*^(fG)^*^(fC)^*^(αlC)^*^(fU)^*^(fC)^*^(αlC)^*^(fG)^*^(fG)^*^(fU)^*^(fU)^*^(fC)^*^(fU)^*^(fG)	—
LNA-FRNA	(fC)^*^(fU)^*^(fG)^*^(fU)^*^(lT)^*^(fG)^*^(fC)^*^(lC)^*^(fU)^*^(fC)^*^(lC)^*^(fG)^*^(fG)^*^(fU)^*^(fU)^*^(fC)^*^(fU)^*^(fG)	>95
2′OMe	(mC)^*^(mU)^*^(mG)^*^(mU)^*^(mU)^*^(mG)^*^(mC)^*^(mC)^*^(mU)^*^(mC)^*^(mC)^*^(mG)^*^(mG)^*^(mU)^*^(mU)^*^(mC)^*^(mU)^*^(mG)	86.5
FRNA	(fC)^*^(fU)^*^(fG)^*^(fU)^*^(fU)^*^(fG)^*^(fC)^*^(fC)^*^(fU)^*^(fC)^*^(fC)^*^(fG)^*^(fG)^*^(fU)^*^(fU)^*^(fC)^*^(fU)^*^(fG)	>85
LNA-2′OMe	(mC)^*^(mU)^*^(mG)^*^(mU)^*^(lT)^*^(mG)^*^(mC)^*^(lC)^*^(mU)^*^(mC)^*^(lC)^*^(mG)^*^(mG)^*^(mU)^*^(mU)^*^(mC)^*^(mU)^*^(mG)	>95

(fX) = 2′FRNA, (mX) = 2′OMe, (lX) = LNA, (nX) = 2′FANA, (αlX) = alphaLNA, ^*^ = phosphorothioate linkage. — = *T_m_* could not be accurately measured. The complementary RNA strand for the series had the following sequence: 5′- CAGAACCGGAGGCAACAG-3′.

#### Synthesis

All oligonucleotide syntheses were performed on an Applied Biosystems 3400 or Expedite DNA Synthesizer using Unylink CPG support (ChemGenes). Modified RNA (FANA, FRNA, and αLNA) phosphoramidites were prepared as 0.11 M solutions in acetonitrile (ACN). LNA 5-Me-Cytidine phosphoramidite was prepared as a 0.11 M in tetrahydrofuran (THF):ACN (50:50 v/v). 5-Ethylthiotetrazole (0.25 M in ACN) was used to activate phosphoramidites for coupling. Detritylations were accomplished with 3% trichloroacetic acid in dichloromethane for 110 s. Capping of failure sequences was achieved with acetic anhydride in THF and 16% N-methylimidazole in THF. Oxidation was done using 0.1 M I_2_ in 1:2:10 pyridine:water:THF. Sulfurization was achieved with 3-(dimethylaminomethylidene)amino-3H-1,2,4-dithiazole-3-thione (DDTT) in ACN.

Coupling times were 30 min for 2′F-RNA and 2′OMe-RNA phosphoramidites and 50 min for LNA phosphoramidites. Deprotection and cleavage from the solid support was accomplished with 3:1:0.2 NH_4_OH:EtOH:DMSO at 65°C for 12 h. Crude oligonucleotides were purified by anion exchange high-performance liquid chromatography on an Agilent 1200 Series Instrument using a Protein-Pak DEAE 5PW column (7.5 × 75 mm) at a flow rate of 1 mL/min. The gradient was 0–24% solution 1 M LiClO_4_ over 30 min at 60°C. Samples were desalted on NAP-25 desalting columns according to manufacturer's protocol. Their masses were confirmed by high-resolution mass spectrometry.

#### UV thermal melting studies

A Varian CARY 100 UV-visible spectrophotometer equipped with a Peltier temperature controller was used to perform the UV thermal melting studies. RNA complement and AON (2 nmol each) were added and dissolved in the appropriate buffer [5 mM sodium phosphate buffer, 140 mM KCl, 1 mM MgCl_2_ (pH 7.4)]. Samples were heated at 95°C for 5 min to ensure denaturation of the single strands. Sample collection was performed between 95°C and 5°C and the sample temperature was lowered at an interval of 0.5°C/min. Nitrogen was used when sample temperature fell below 15°C. Absorbance was measured at 260 nm and the thermal melting temperature (*T_m_*) were determined by calculating the midpoint of dissociation curves by first derivatives.

### In vitro assessment

Human control KM155.C25 myoblasts were plated in gelatin-coated 6-well plates at a density of 100,000 cells/well and grown in skeletal muscle cell growth medium KIT (Promocell, Heidelberg, Germany) supplemented with 15% fetal bovine serum and 50 μL/mg gentamicin (ThermoFisher Scientific, Waltham, MA) at 37°C with 5% CO_2_ in a humidified incubator. Once cells were 90% confluent, differentiation medium containing Dulbecco's modified Eagle's medium (low glucose, pyruvate, no glutamine, no phenol red, ThermoFisher Scientific) supplemented with 50 μL/mg gentamicin, 2% Glutamax (ThermoFisher Scientific), and 1% d-glucose (Sigma Aldrich, Saint Louis, MI) was added to the cells. Cells were differentiated into myotubes for 3 days before gymnosis experiments.

Gymnosis (free uptake) was performed in duplo in myotubes at concentrations of 125, 250, and 500 nM of AON. As a negative control a 2′OMe-PS AON was used targeting exon 47 of the *DMD* transcript (5′-UCUUGCUCUUCUGGGCUU-3′). To resemble physiological conditions, CaCl_2_ was added to the differentiation medium to a final concentration of 9 mM. The AONs were diluted in the CaCl_2_-rich differentiation medium to the desired concentration, after which the mix was added to the cells followed by incubation at 37°C for 72 h. Afterward, cells were harvested using TriSure reagent (Meridian Bioscience, OH) and stored at −80°C.

### Animal studies

Male hDMDdel52/*mdx* (Tg(DMD*)del52Lumc) mice were bred in the animal facility of the Leiden University Medical Center. Mice were housed in individually ventilated cages at 20.5°C with 12 h of light/dark cycles. Mice had *ad libitum* access to water and standard RM3 chow (SDS, Essex, United Kingdom) throughout the experiment. All animal experiments were approved by the Animal Welfare Body of the Leiden University Medical Center.

#### Intramuscular treatment

To test the efficiency of the AONs to induce exon 53 skipping *in vivo*, the top three AON candidates (FRNA, LNA-FRNA, and LNA-2′OMe) and a positive control (2′OMe) were intramuscularly injected in the triceps and gastrocnemius of three male hDMDdel52/*mdx* mice (20 μg of AON in 40 μL volume per muscle) on 2 consecutive days. Per mouse, the four AONs were randomly divided over the muscles. 10 days after the last injection, mice were sacrificed, and the triceps and gastrocnemius muscles were isolated, snap-frozen in isopentane cooled in liquid nitrogen, and stored at −80°C.

#### Systemic treatment

Male hDMDdel52/*mdx* mice (Saline, *n* = 6; 2′OMe, *n* = 5; LNA-2′OMe, *n* = 6; FRNA, *n* = 5; LNA-FRNA, *n* = 7) were randomly divided over the different experimental groups at 4 weeks of age. Mice received weekly subcutaneous injections of 50 mg/kg of AON in 100 μL saline (0.9% NaCl; Fresenius Kabi, Bad Homburg, Germany) for the duration of 6 weeks. A control group of hDMDdel52/*mdx* mice was included, which received weekly subcutaneous injections of 100 μL saline. Bodyweight was recorded once a week for the duration of the experiment. One week after the last injection, blood was taken via a small cut in the tail vein and collected in heparin-coated microvette tubes (Sarstedt, Numbrecht, Germany) to assess creatine kinase (CK) levels and markers for liver and kidney function.

Samples were kept on ice after which they were centrifuged at 4°C for 5 min at 13,000 rpm. Levels of CK, urea, glutamic oxaloacetic transaminase (GOT, also known as aspartate aminotransferase), glutamate pyruvate transaminase (GPT, also known as alanine aminotransferase), and alkaline phosphates (ALP) were assessed using Reflotron strips and the Reflotron Sprint system (Roche Diagnostics, Basel, Switzerland). Thereafter, mice were sacrificed and the gastrocnemius, triceps, tibialis anterior, diaphragm, and heart were isolated, snap-frozen in isopentane cooled in liquid nitrogen, and then stored at −80°C.

### RNA isolation

The cell/TriSure mix was thawed on ice and homogenized by vortexing the samples. Tissues were transferred to zirconium bead prefilled tubes (OPS Diagnostics, Lebanon) to which TriSure was added. Tissues were homogenized using the MagNA Lyser (Roche Diagnostics). Total RNA was isolated using the TriSure protocol. Samples from the systemic treatment experiment were further purified using the NucleoSpin RNA kit (Macherey-Nagel, Düren, Germany), according to the manufacturer's instructions. RNA concentration was determined using the Nanodrop (Thermo Fisher Scientific, Waltham, MA) and stored at −80°C.

### Complementary DNA synthesis

Complementary DNA (cDNA) was made by incubating 1 μg total RNA with random hexamer primers and deoxynucleotide triphosphates (dNTPs) (Thermo Fisher Scientific) for 5 min at 70°C to melt out RNA secondary structures. Thereafter, Tetro reverse transcriptase (RT), 5 × reaction buffer (Meridian Bioscience), and recombinant RNasin Ribonuclease inhibitor (Promega, Leiden, The Netherlands) were added and the samples were incubated for 10 min at 25°C, 1 h at 42°C, 5 min at 85°C, and cooled down to room temperature. The cDNA was stored at 4°C.

For the systemic experiments involving a single RT-PCR (see section ‘RT-PCR analysis for exon skip assessment’), the incubation step to melt out RNA secondary structures before cDNA synthesis was done at either 70°C or 95°C. Then, Moloney Murine Leukemia Virus RT, 5 × reaction buffer (Promega), and RNAsin Ribonuclease inhibitor were added and the samples were incubated for 10 min at 25°C, 1 h at 42°C, and 10 min at 70°C and cooled down to room temperature. The cDNA was stored at 4°C.

The effect of 5 min of incubation at 70°C or 95°C on RNA integrity was assessed with the RNA Nano kit on the Agilent 2100 Bioanalyzer using 1 μg total RNA from the tibialis anterior in the absence of random hexamer primers and dNTPs. Per temperature condition, two RNA samples of the saline-treated group and one RNA sample of each AON treatment group were used.

### RT-PCR analysis for exon skip assessment

#### Nested RT-PCR

RT-PCR analysis was performed in the following manner: cDNA or RT-PCR product was mixed with Taq DNA polymerase (Roche), PCR buffer with MgCl_2_ (Roche), dNTPs, and forward and reverse primers. The first RT-PCR contained 15% (3 μL) of cDNA and was carried out for 25 cycles at 94°C (40s), 60°C (40s), and 72°C (3 min) with primers targeting exon 50 and exon 55 (*in vitro*) or exon 48 and exon 57 (*in vivo*). A second RT-PCR round was done using 7.5% (1.5 μL) RT-PCR1 product and primers targeting exon 52 and exon 54 (*in vitro*) and exon 49 and exon 54 (*in vivo*), for 32 cycles at 94°C (40s), 60°C (40s), and 72°C (90s). PCR products were analyzed on 2% agarose gels. PCR products for the systemic experiments were semiquantified with the DNA1000 kit on the Agilent 2100 Bioanalyzer (Agilent, Amstelveen, The Netherlands).

To assess the interference of LNA-modified AONs in the RT-PCR, 10 or 20 ng of AON was added to either the first or both RT-PCRs.

#### Single RT-PCR

The RT-PCR contained 12.5% (2.5 μL) of cDNA, mixed with DreamTaq DNA polymerase, 10 × DreamTaq buffer (Thermo Fisher Scientific), dNTPs, and forward (Exon 49) and reverse (Exon 54) primers. The RT-PCR ran for 36 cycles at 95°C (40s), 48°C (40s), and 72°C (90s). PCR products were analyzed on 2% agarose gels and semiquantified with the DNA1000 kit on the Agilent 2100 Bioanalyzer.

### DMD transcript level analysis

To determine the effect of AON treatment on *DMD* transcript levels, RT-quantitative PCR (qPCR) was performed. The cDNA was made with RNA from the gastrocnemius that was incubated at either 70°C or 95°C before RT.

RT-qPCR was performed with the LightCycler480 system (Roche Diagnostics). One percent of cDNA was used per reaction and each cDNA sample was tested in triplicate in the presence of SensiMix SYBR Hi-ROX kit (Meridian Bioscience) and primers targeting exon 3–4, exon 46–47, or exon 78-untranslated region (UTR) junction. Expression values were analyzed with the LinReg PCR software (version 2018.0) [19], normalized to the housekeeping gene *Gapdh*, after which the ratio between the expression values of exon 3–4 junction of the saline-treated group of 70°C pretreated RNA and each of the other conditions was calculated.

### Protein isolation and western blot

Dystrophin protein levels were assessed by western blot of the gastrocnemius, tibialis anterior, and heart. Protein lysates were generated by homogenizing muscle in isolation buffer [20% sodium dodecyl sulfate (SDS) in 0.1 M Tris-HCl (pH 6.8)] in zirconium bead prefilled tubes with the MagNA Lyser. Protein lysates were denatured at 95°C, after which the protein concentration was determined with the Pierce BCA protein assay kit (Thermo Fisher Scientific) according to the manufacturer's instructions. Protein lysates from the corresponding muscles of a hDMD/*mdx* mouse (which carries the intact human *DMD* gene) were used to generate a concentration curve for dystrophin.

Protein samples (35 μg of total protein) were prepared in Laemmli buffer (2% SDS, 10% glycerol, 60 mM Tris-HCl [pH6.8]) and supplemented with 2% β-mercaptoethanol and 0.01% bromophenol blue and incubated for 5 min at 95°C. Thereafter, protein samples were loaded on a Criterion XT 3%–8% Tris-Acetate gel in XT Tricine running buffer (Bio-Rad, Basel, Switzerland) and were run for 1 h at 75 V and 1 h at 150 V. Proteins were transferred onto a 0.2 μm Nitrocellulose membrane with the Trans-Blot Turbo Transfer system (Bio-Rad).

Membranes were blocked in 5% nonfat dried milk powder (Sigma-Aldrich) in Tris-buffered saline for 1 h. Next, blots were incubated with the primary antibodies Ab154168 (1:2,000; Abcam, Amsterdam, The Netherlands) to detect human dystrophin, and 66895-1-Ig (1:1,000; Proteintech, Wuhan, China) and to detect α-actinin as a loading control, in Takara Western BLoT Immunobooster (Takara Bio, Inc., France). The following day, blots were washed to remove unbound primary antibodies and then incubated with the secondary antibodies donkey-α-Rabbit IRDye-800CW and donkey-α-Mouse IRDye-680RD (Li-Cor, Lincoln). The membrane was visualized with the Odyssey CLx infrared imaging system and software (Li-Cor).

### Peptide nucleic acid hybridization assay

AON levels were detected in the tibialis anterior using the peptide nucleic acid (PNA) assay as previously described [20]. In brief, tissues were homogenized in QuantiGene™ Homogenizing Solution (Invitrogen) using the TissueLyser II bead mill (Qiagen) followed by treatment with 3 M potassium chloride to precipitate SDS. Following centrifugation, supernatants were transferred to 96-well PCR plate and hybridized to Alexa 488 labeled PNA probe (PNAbio, Inc.) designed to be fully complementary to the AON sequence. The samples were then run on the DNAPac PA100 anion exchange column (Thermo Fisher Scientific) and detected by a 1260 FLD fluorescent detector. The amount of AON in solution was quantified based on the integrated peak area of the bound PNA probe by extrapolation from a standard curve run with known amounts of AON.

### Statistics

Data analyses were performed with GraphPad Prism (GraphPad Software, San Diego, CA, version 8.1.1) or SPSS (IBM SPSS Statistics for Windows, Version 25.0.; IBM Corp., Armonk, NY). Values are presented as means ± standard deviation. *P* < 0.05 was considered statistically significant.

Outliers in plasma markers and exon 53 skip percentages were determined with the ROUT method (*Q* = 1%) in GraphPad Prism. Outliers are visualized in the graphs but were removed for further analysis or descriptive statistics. Bodyweight was analyzed with a mixed model for linear regression in SPSS. A Shapiro-Wilk test was performed to test the normality of the plasma markers data. A Kruskal-Wallis test and the Dunn's multiple comparison test were performed to compare between the different treatment groups for the CK, GOT, and GPT data. The ALP and urea data were analyzed with a one-way analysis of variance followed by a Tukey's multiple comparison test.

## Results

### In vitro exon 53 skipping in human myotubes

**FIG. 1.**
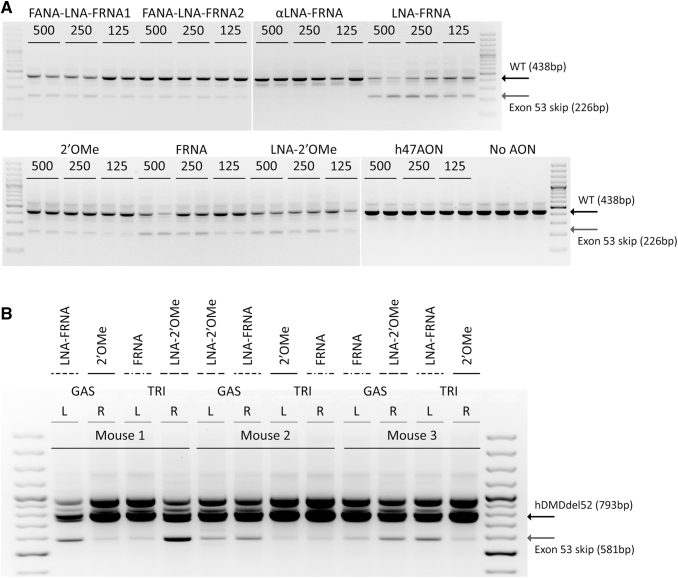
*In vitro* and *in vivo* validation of AON functionality. **(A)** RT-PCR analysis of exon 53 skipping in human myotubes after gymnosis with 125, 250, and 500 nM of AON. As a negative control, cells received a 2′OMe-PS AON targeting exon 47 or were left untreated (no AON). The WT product contains exon 52–54 (438 bp), while the exon 53 skip product only contains exon 52 and 54 (226 bp). **(B)** RT-PCR analysis of exon 53 skipping in hDMDdel52/*mdx* mice upon local delivery of human specific exon 53-targetting AONs. Three mice received an intramuscular injection of the 2′OMe, LNA-2′OMe, FRNA, or LNA-FRNA AON in their L or R GAS or TRI. The hDMDdel52 product contains exons 49–51 and 53–54 (793 bp), while the exon 53 skip product contains exons 49–51 and 54 (581 bp). The band at ∼1,000 bp is a cryptic splicing event previously described (Veltrop, 2018). AON, antisense oligonucleotide; GAS, gastrocnemius; L, left; PS, phosphorothioate; R, right; RT-PCR, reverse transcriptase polymerase chain reaction; TRI, triceps; WT, wild-type.

To assess the efficiency of the AONs to induce exon 53 skipping, 125, 250, or 500 nM AON were delivered via gymnosis into myotubes. No exon 53 skipping was observed after treatment with the αLNA-FRNA-modified AON (Fig. 1A). The FANA-LNA-FRNA1 and FANA-LNA-FRNA2 AONs resulted in low amounts of exon 53 skipping. Treatment with the LNA-FRNA-, FRNA-, and LNA-2′OMe-modified AONs resulted in the most abundant exon 53 skipping and were therefore selected for further *in vivo* testing with the 2′OMe-modified AON as a control.

### In vivo validation of AON-mediated exon 53 skipping upon intramuscular treatment

To study the functionality of our top three AON candidates selected from the *in vitro* experiment, we tested the AONs in the hDMDdel52/*mdx* mouse upon local delivery. Due to the partial deletion of exon 52 in the human *DMD* gene, that abolishes inclusion of exon 52 into the mRNA, human-specific AONs targeting exon 53 are expected to restore the disrupted reading frame. Mice received intramuscular injections of AONs on 2 consecutive days in the triceps and gastrocnemius. Exon 53 skip levels were assessed with an RT-PCR and visualized on an agarose gel (Fig. 1B). In the muscles that were treated with the 2′OMe and the FRNA AONs, we observed low levels of exon 53 skip product. In contrast, treatment with the LNA-2′OMe and the LNA-FRNA AON resulted in high amounts of exon 53 skip product.

### Evaluation of exon 53 skipping after systemic treatment with human-specific AONs

#### Systemic treatment of AONs in hDMDdel52/*mdx* mice was well tolerated

Encouraged by the observed exon 53 skip efficiency after intramuscular injections, we continued with a systemic treatment study utilizing the same AONs. Male hDMDdel52/*mdx* were treated with weekly subcutaneous injections of 50 mg/kg AON or saline for 6 weeks, starting at 4 weeks of age (*n* = 5–7 per group). The bodyweight was recorded once a week for the duration of the experiment but did not statistically differ between the treatment groups over time (Supplementary Fig. S1A).

One week after the last treatment, blood was collected and markers for muscle fiber integrity as well as for liver and kidney function were assessed for safety and tolerability of the AONs. CK is a marker for muscle damage and is highly increased in DMD patients and *mdx* mice. No difference in CK levels was observed between the different treatment groups (Supplementary Fig. S1B). GOT and GPT are enzymes that are elevated in blood upon muscle as well as liver damage [21]. GOT and GPT levels were similar in the different treatment groups (Supplementary Fig. S1C, D). No statistical difference in urea (marker for kidney function) and ALP (marker for liver function) levels were detected between the treatment groups (Supplementary Fig. S1E, F).

#### Exon skip levels after systemic treatment

**FIG. 2.**
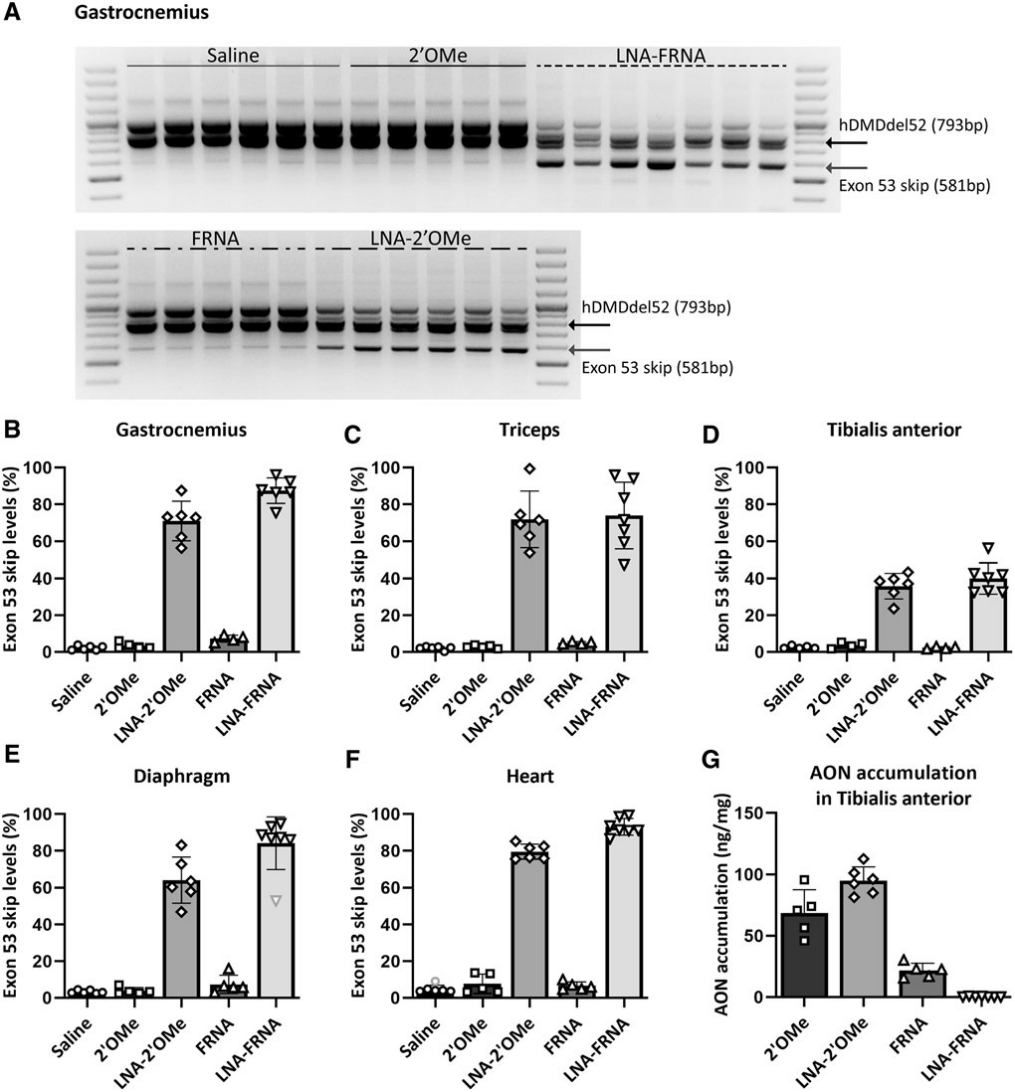
Exon 53 skip assessment and AON accumulation after systemic treatment with AONs in hDMDdel52/*mdx* mice. **(A)** RT-PCR analysis of exon 53 skipping in the GAS of hDMDdel52/*mdx* mice upon systemic treatment with human specific exon 53-targetting AONs. The hDMDdel52 product contains exons 49–51 and 53–54 (793 bp) while the exon 53 skip product contains exons 49–51 and 54 (581 bp). **(B–F)** Semiquantified exon 53 skip levels in the GAS, TRI, tibialis anterior, diaphragm, and heart. Outliers are visualized in *light gray*. **(G)** AON accumulation levels in the tibialis anterior.

Exon 53 skip levels were assessed with a nested RT-PCR in skeletal muscles (gastrocnemius, triceps, tibialis anterior, and diaphragm) and heart (Fig. 2A–F). We observed very low levels of exon 53 skipping in saline-treated mice, which can be due to low level of spontaneous, frame restoring exon skipping and is also observed in DMD patients [22]. Treatment with the 2′OMe AON resulted in low levels of exon 53 skip (<4% in skeletal muscles and 7.7% in heart). Treatment with the FRNA AON resulted in slightly higher exon 53 skipping in the gastrocnemius, diaphragm, triceps, and heart (up to 7.4% in skeletal muscle and 6.6% in heart), while exon 53 skip levels in the tibialis anterior were comparable to the spontaneous skip level in saline-treated mice (FRNA: 2.4% ± 0.4%; saline: 2.6% ± 0.8%). The most pronounced exon 53 skip levels were observed in skeletal muscles and heart of mice treated with the LNA-2′OMe or the LNA-FRNA AONs with levels of 35.8% and 39.9% in the tibialis anterior, 64.0%–71.9% and 74.0%–89.5% in other skeletal muscles, and 79.5% and 93.0% for heart, respectively (Fig. 2A–F).

AON accumulation was determined for the tibialis anterior. The 2′OMe and LNA-2′OMe AONs were most abundantly present after systemic treatment, while the concentration of FRNA AONs was much lower. Notably, LNA-FRNA AONs could not be detected in the tibialis anterior (Fig. 2G), despite the high exon skipping levels observed.

#### Limited restoration of dystrophin expression after AON treatment

**FIG. 3.**
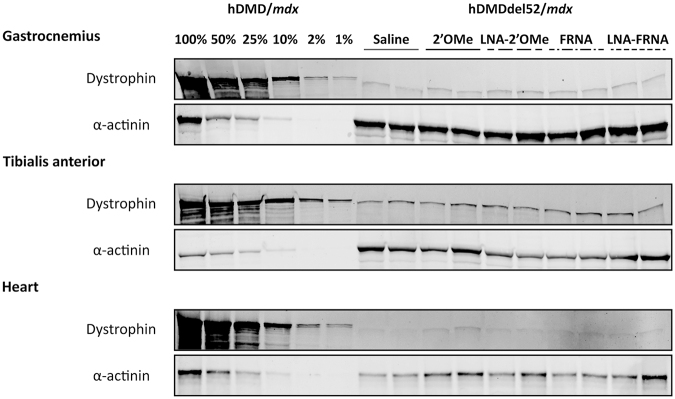
Western blot analysis of dystrophin protein levels after systemic treatment with human specific exon 53-targeting AONs in hDMDdel52*/mdx* mice. Protein lysates from GAS, tibialis anterior, and heart of a hDMD/*mdx* mouse (which carries the intact human *DMD* gene) were used as a positive control for WT dystrophin levels. α-actinin was used as a loading control.

Since high levels of exon 53 skip were observed after treatment with the LNA-2′OMe and the LNA-FRNA AONs compared to the 2′OMe AON, we assessed whether this resulted in dystrophin restoration by western blot analysis. We observed very low levels of dystrophin protein (<1%) in the saline-treated group. Unexpectedly, we did not observe a visual increase in the dystrophin protein amounts in the AON-treated groups in the gastrocnemius, tibialis, and heart (Fig. 3).

#### LNA interference resulted in overestimation of exon skip levels

**FIG. 4.**
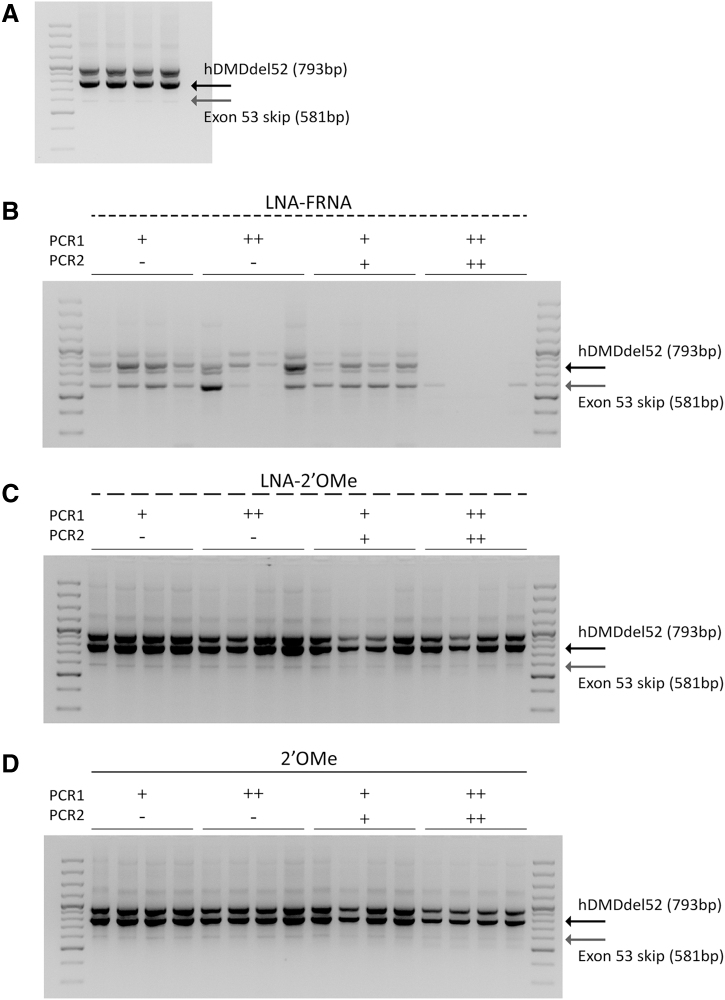
The presence of AONs in the RT-PCR influences the efficiency, in which the hDMDdel52 product is being amplified. **(A)** RT-PCR analysis of exon 53 skipping in four GAS samples from hDMDdel52/*mdx* mice treated with saline. Low levels of spontaneous exon 53 skip can be observed when no AON is being added to the first or both polymerase chain reaction rounds (−). The influence of AON presence in the RT-PCR was assessed in these samples by adding 10 (+) or 20 ng (++) of **(B)** LNA-FRNA, **(C)** LNA-2′OMe, or **(D)** 2′OMe AON to the first or both RT-PCR rounds. The hDMDdel52 product contains exons 49–51 and 53–54 (793 bp), while the exon 53 skip product contains exons 49–51 and 54 (581 bp).

To investigate potential causes for the discrepancies observed between high exon skip levels after treatment with LNA-2′OMe and LNA-FRNA AONs and the absence of treatment effects on dystrophin restoration, we assessed whether the increased binding affinity of the LNA-modified AONs to the target exon interfered with the amplification of the hDMDdel52 product in the RT-PCR. To do so, we added 10 or 20 ng of LNA-2′OMe, LNA-FRNA, or 2′OMe, as a control, in either the first or both PCR steps of the nested RT-PCR of four saline-treated gastrocnemius samples, in which we observe low levels of spontaneous exon 53 skip product (Fig. 4A). The addition of 10 ng LNA-FRNA AON to either the first or both RT-PCR rounds resulted in an increase in the exon 53 skip product and a decrease in the hDMDdel52 product.

When 20 ng LNA-FRNA AON was added to the reaction, the effect on the amplification of both products was even more pronounced, resulting in the absence of the hDMDdel52 unskipped and exon 53 skip product when added to both RT-PCR rounds (Fig. 4B). The addition of LNA-2′OMe or 2′OMe AONs did not affect the amplification of the PCR products, as only a subtle decrease was observed for the hDMDdel52 product when 20 ng of LNA-2′OMe or 2′OMe AON was added to both RT-PCRs (Fig. 4C, D).

Although we confirmed the effect of AON interference in the RT-PCR, it is more likely that the presence of LNA-modified AONs itself already influences cDNA synthesis. Therefore, we aimed to remove RNA structures before cDNA synthesis and optimize the RT-PCR. To melt out RNA secondary structures, it is recommended to incubate RNA samples at 70°C in the presence of primers and dNTPs before cDNA synthesis. Since the LNA modification increases the affinity of the AON for the target RNA, we evaluated the thermal affinities of the AONs to the complementary RNA. Indeed, we measured that the thermal affinity of the LNA-FRNA or LNA-2′OMe AONs was >95°C (Table 1).

Therefore, we increased the RNA incubation temperature to 95°C to melt the LNA-modified AONs from the mRNA before cDNA synthesis. In addition, we optimized our RT-PCR to a single PCR since nested RT-PCR is known to overamplify smaller fragments, in this case, the exon 53 skip products. To ensure that the RNA integrity was not impacted, we ran an RNA Nano chip, which confirmed that the RNA integrity number was sufficient for RT-PCR analysis before and after incubation at higher temperatures (>5, 8.4 ± 0.3 in the samples incubated at 70°C and 6.3 ± 0.2 in the samples incubated at 95°C).

**FIG. 5.**
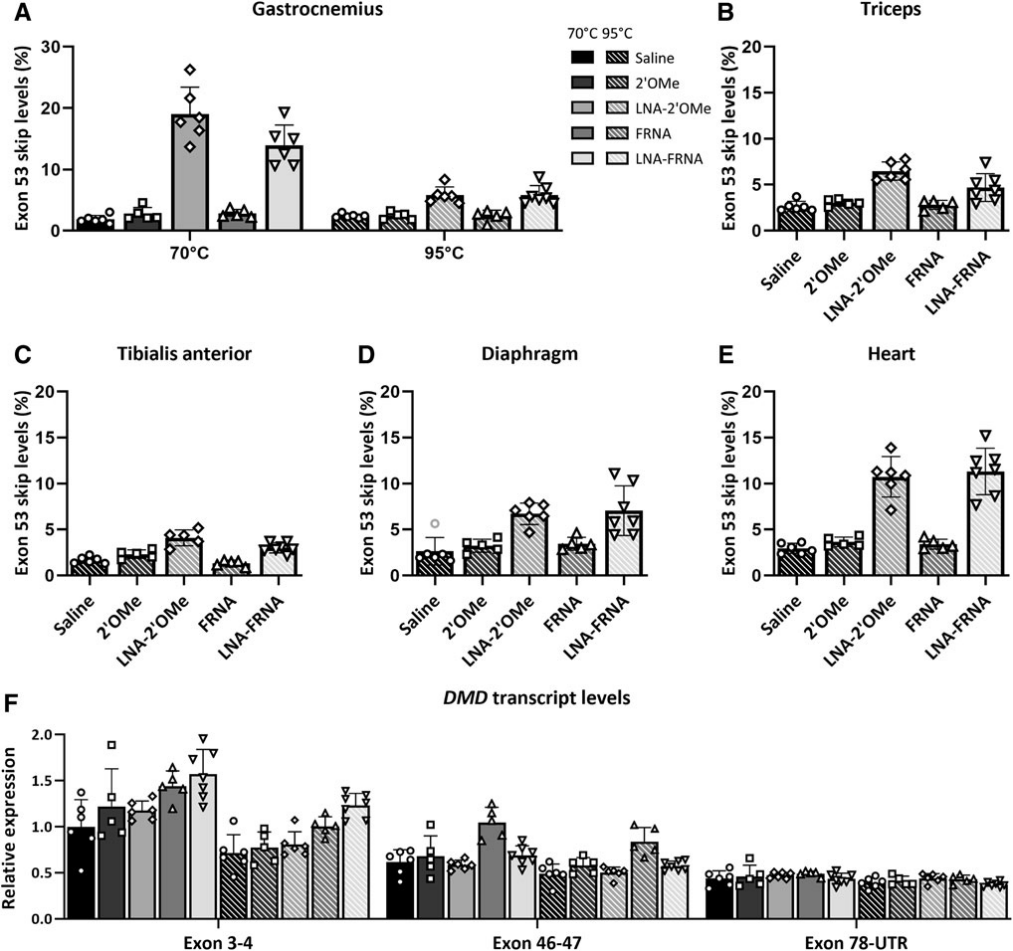
Exon 53 skip assessment with a single RT-PCR in skeletal muscles and heart and *DMD* transcript levels assessment in the GAS of hDMDdel52/*mdx* mice after systemic AON treatment. RT-PCR analysis with cDNA synthesized after RNA incubation at **(A)** 70°C and 95°C in GAS or 95°C in **(B)** TRI, **(C)** Tibialis anterior, **(D)** Diaphragm, and **(E)** Heart. **(F)** *DMD* transcript levels at exon 3–4, exon 46–47, and exon 78-UTR exon junctions in the GAS with cDNA synthesized after RNA incubation at 70°C or 95°C.

We observed that in a single RT-PCR with cDNA made from the gastrocnemius RNA samples, which were incubated at 70°C before cDNA synthesis, the amount of exon skip levels was drastically decreased for the LNA-2′OMe and LNA-FRNA AONs to 19.0% ± 4.4% and 13.9% ± 3.3%, respectively (Fig. 5 and Supplementary Fig. S2A). When we increased the RNA incubation temperature to 95°C before cDNA synthesis, followed by a single RT-PCR round, we observed an even further decrease to 5.8% ± 1.4% and 5.8% ± 1.5% of exon 53 skipping in the gastrocnemius of mice treated with the LNA-2′OMe and the LNA-FRNA AONs (Fig. 5 and Supplementary Fig. S2B).

A similar reduction in exon 53 skip level was observed in the triceps, tibialis anterior, diaphragm, and heart with RNA incubated at 95°C before cDNA synthesis. We did not observe an effect of RT-PCR type or RNA incubation temperature on exon 53 skip levels in skeletal muscles or heart of mice treated with the 2′OMe AON, and only a slight decrease in FRNA AON-treated samples (Fig. 5A–E).

To assess whether AON treatment influenced *DMD* transcript levels, we performed a RT-qPCR at different exon-exon junctions in the gastrocnemius of hDMDdel52/*mdx* mice. We observed a trend toward increase in *DMD* transcript levels at the exon 3–4 junction in all AON-treated groups compared to the saline-treated group. At the exon 46–47 and exon 78-UTR junction, all treatment groups had comparable levels. We also assessed the *DMD* transcript levels with cDNA generated from RNA incubated at 95°C before cDNA synthesis. We observed that the 95°C RNA pretreatment resulted in overall lower exon 3–4 *DMD* transcript levels compared to the 70°C pretreatment although the pattern in which AON-treatment affected *DMD* transcript levels remained the same. At the exon 46–47 and 78-UTR junctions, no decrease was observed (Fig. 5F).

## Discussion

AON-mediated therapies are promising to restore dystrophin production in DMD patients. In this study, we compared the efficacy of different chemically modified human-specific AONs targeting exon 53 in the hDMDdel52/*mdx* mouse model.

We initially observed that treatment with the LNA-2′OMe and LNA-FRNA modified AONs consistently induced high exon 53 skip levels both in human myotubes as well as after local and systemic treatment in hDMDdel52/*mdx* mice, but that this did not translate into increased dystrophin protein levels in skeletal muscles and heart. Several factors may be responsible for the discrepancy between treatment effects observed on RNA and protein level. First, our systemic study was designed with a focus on examination of exon 53 skipping levels rather than protein restoration. Mice were treated for 6 weeks and were sacrificed 1 week after the last injection when exon skipping levels are at a maximum, while dystrophin protein levels peak between 4 and 12 weeks post-treatment, as observed previously in pharmacodynamic studies of 2′OMe-PS AONs [23].

However, given the unprecedented high exon 53 skipping levels observed in skeletal muscles and heart for the LNA-2′OMe- and the LNA-FRNA AON-treated animals, we anticipated to see an earlier treatment effect on protein level.

Second, there might have been a preferential overamplification of the shorter, skipped transcript compared to the longer, unskipped transcript. As we assessed exon 53 skip levels with a nested RT-PCR for 25 + 32 cycles, even a minor increase in amplification efficiency of the skipped product can lead to a drastic overestimation of skipping efficiency. This overamplification would, however, apply to all the samples of the different treatment groups. Thus, while we believe it is likely that some overamplification of the skipped product has occurred, this does not explain why we observed similar protein levels in the different treatment groups when observing much higher exon 53 skip levels for the LNA-FRNA- and LNA-2′OMe AON-treated groups compared to the 2′OMe- and FRNA AON-treated groups.

Since the LNA modifications greatly increase the binding affinity of the AONs to the target RNA and some of the AONs might remain bound to the RNA during RNA isolation, we hypothesized that the LNA-2′OMe and the LNA-FRNA AONs were hybridizing to the unskipped fragments and therefore interfering during cDNA synthesis and PCR amplification. Indeed, the high hybridization affinity of the LNA modified AONs (*T*_m_ > 95°C) interfered with cDNA synthesis and could be (partially) prevented by incubating the RNA samples at 95°C instead of the standard 70°C incubation to remove RNA secondary structures. Furthermore, the addition of LNA-FRNA AON to the RT-PCR almost completely prevented amplification of the unskipped product and resulted in artificial overamplification of the exon 53 skip product while the effect of the LNA-2′OMe AON on the amplification of the unskipped product was smaller but still detectable.

The difference in how addition of LNA-FRNA or LNA-2′OMe influenced the RT-PCR could potentially be explained by differences in AON abundance in the tissues, in which LNA 2′OMe AON levels exceeded those of the LNA-FRNA AONs. Notably, we observed that the LNA-FRNA AON was not detectable in the tibialis anterior, whereas the LNA-2′OMe AONs were, despite similar exon skipping levels. A possible explanation is that the LNA-FRNA has some secondary structure that interfered with the binding to the probe.

Our finding that the LNA-FRNA and 2′OMePS-LNA AONs interfere with cDNA synthesis and RT-PCR analysis has not only implications for splice modulating AONs but also for RNase H AON approaches, aiming at achieving knockdown of target transcript. Like with our approach, RNase H AON interference with cDNA synthesis and PCR amplification can artificially suggest that AONs are effective in achieving knockdown. To our knowledge this LNA modified AON interference has not previously been reported when studying therapeutic oligonucleotides and only a few articles discuss efficacy of LNA-2′OMe and LNA-FRNA splice modulating AONs. For instance, LNA-FRNA-PS AONs have been evaluated *in vitro* in *mdx* myotubes for exon 23 skipping of the murine *Dmd* transcript and were observed to result in higher exon 23 skipping efficiency than 2′OMe-PS, FRNA-PS, and 2′OMe-FRNA-PS AONs [24].

Since this study is only based on *in vitro* assessment of exon 23 skip levels, it is hard to tell whether the observed exon 23 skip efficiency with the LNA-FRNA-PS AONs could have been caused by the LNA interference we observed in our study. A recent article by van Deutekom and colleagues studied exon 51 skipping after treatment with different 2′OMe-PS AONs with 5-methylcytosines throughout the AON and different LNA patterns in the hDMDdel52/*mdx* mouse model for a duration of 13 weeks [25]. They identified an alternative target site to the drisapersen/eteplirsen target site and identified three AONs that resulted in efficient exon 51 skipping as well as dystrophin restoration. Since the observed exon 51 skipping translates into protein restoration, it does not appear that their LNA-2′OMe AONs caused any interference within the cDNA synthesis and during the ddPCR analysis to assess exon 51 skip levels.

AONs modified with LNA-2′OMe-PS have also been studied for the treatment of myotonic dystrophy type 1 (DM1). DM1 is caused by a CTG repeat expansion in the *DMPK* gene, which results in a toxic gain-of-function mRNA that sequesters MBNL1, an ubiquitously expressed splicing factor, in the nucleus resulting in disordered MBNL1 autoregulation of its pre-mRNA splicing. MBNL1 function can be restored with exon 7 skipping AONs. Christou and colleagues evaluated LNA-2′OMe modified AONs on exon 7 exclusion of *Mbnl1* and the downstream effect of alternative *Mbnl1* splicing on exon 22 inclusion of *Serca1* in mouse myoblasts and after intramuscular and systemic treatment in HAS^LR^ mice [26]. While they initially observed efficient exon 7 exclusion of *Mbnl1 in vitro* and partial reversal of the misregulated splicing of *Mbnl1* and *Serca1* with intramuscular treatment, they did not observe exon 22 inclusion of *Serca1* after systemic treatment.

However, while they show that the LNA-2′OMe AONs accumulate in skeletal muscles and heart, they do not mention the effect of AON treatment on exon 7 skipping of *Mbln1,* which raises the question whether these AONs were able to induce exon 7 skipping of *Mbnl1* or if there was a discrepancy between the treatment effect on alternative splicing of *Mbln1* and *Serca1* [26].

Notably, in the first two studies, the LNA modifications were primarily located around the 5′ and 3′ end of the AON, while in the last study, the LNA modifications were more evenly spread across the AONs, whereas in our study the LNA modifications are located in the core of the AONs. The position of the LNA modification within the AON might greatly influence the ability to which the LNA modified AON are able to interfere during PCR analysis. Interestingly, LNA/DNA mixmers have been used in assays to block amplification of a wild-type DNA fragment, allowing for the amplification and detection of single-point mutant DNA fragments [27,28] and, in a recent study, the amplification selectivity was assessed by adapting the LNA profile of LNA/DNA mixmers [29].

Furthermore, different chemically modified DNA oligos have been utilized in cell-free systems as well as *in vitro* to block viral reverse transcription of viral RNA either by physically blocking initiation or elongation of cDNA synthesis, by inducing RNase H-mediated cleavage or by direct binding to the RT [30–32].

Given that treatment of hDMDdel52/*mdx* mice with the LNA-FRNA and 2′OMe AONs resulted in higher exon skip levels in skeletal muscles and heart compared to the 2′OMe AON, even after optimization of the cDNA synthesis and RT-PCR analysis to reduce the LNA-modified AON interference, further long-term studies are necessary to evaluate whether these AONs or other permutations of these chimeric AONs can result in dystrophin restoration. Furthermore, based on the study identifying an alternative target size for exon 51 skipping in hDMDdel52/*mdx* [25], alternative target sites for potentially more optimal exon 53 skipping should be considered.

In conclusion, our results show that 2′OMePS-LNA- and LNA-FRNA-modified AONs may have the potential to increase exon-skipping efficacy in a splice modulating approach in skeletal muscles and heart. However, care must be taken during RNA analysis as binding of these and other high-affinity AONs to unskipped transcripts can result in an artificial amplification of efficacy. This likely also applies to applications using RNase H AONs where qPCR is used to assess efficacy.

## Supplementary Material

Supplemental data

Supplemental data
