# Supplementary material for: Challenges of Assessing Exon 53 Skipping of the Human DMD Transcript with Locked Nucleic Acid-Modified Antisense Oligonucleotides in a Mouse Model for Duchenne Muscular Dystrophy
Source: Nucleic Acid Ther. 2023 Nov 24;33(6):348–60. doi: 10.1089/nat.2023.0038 (PMC10698779; doi:10.1089/nat.2023.0038)
Supplement: Supplemental data [file Suppl_FigS1.pdf]

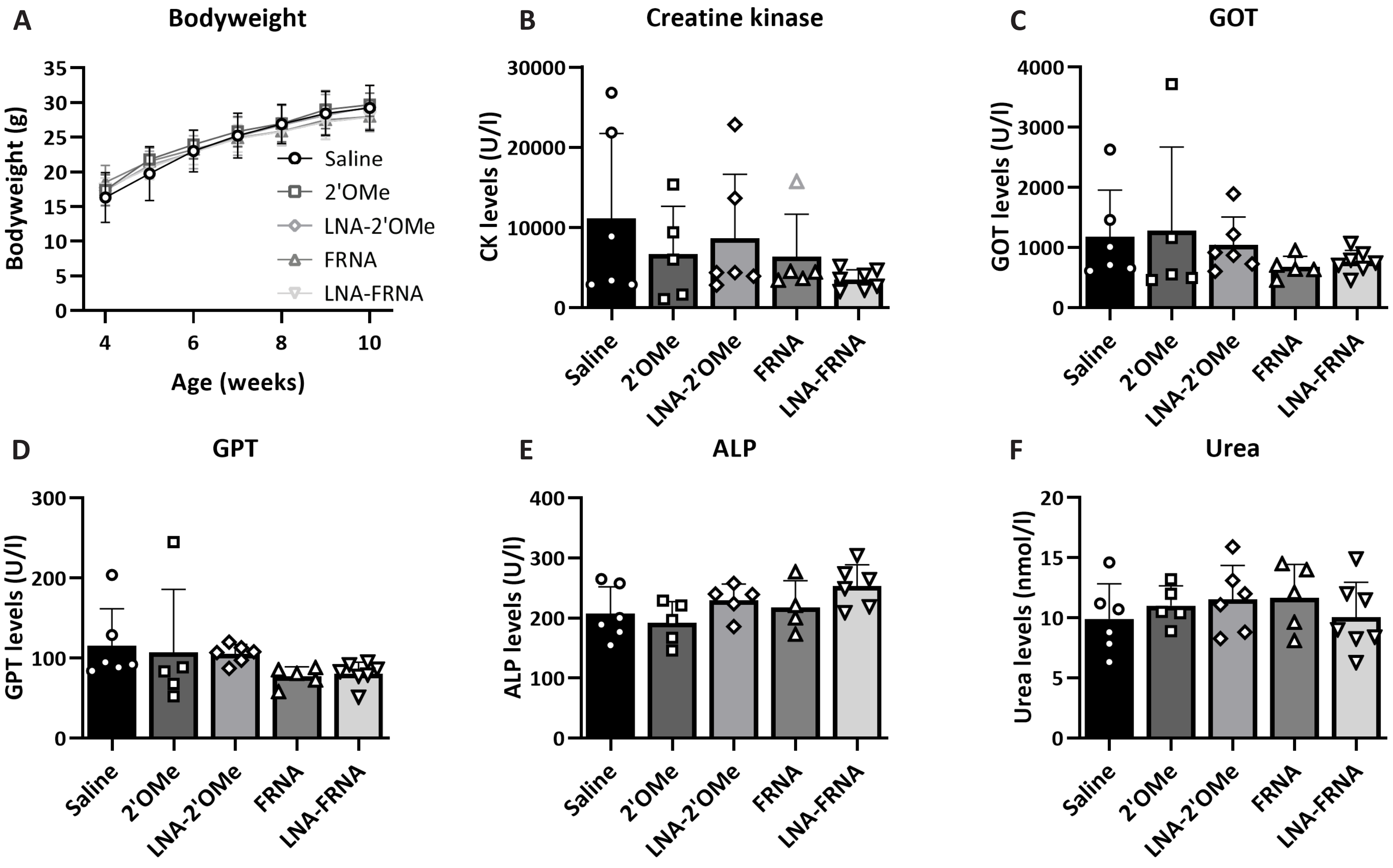

Supplementary Figure 1. Tolerability assessment of AON treatment on bodyweight and plasma protein markers. **(A)** Bodyweight over time **(B)** Creatine kinase **(C)** Glutamic oxaloacetic transaminase **(D)** Glutamate pyruvate transaminase **(E)** Alkaline phosphatase **(F)** Urea.
