# Supplementary material for: Challenges of Assessing Exon 53 Skipping of the Human DMD Transcript with Locked Nucleic Acid-Modified Antisense Oligonucleotides in a Mouse Model for Duchenne Muscular Dystrophy
Source: Nucleic Acid Ther. 2023 Nov 24;33(6):348–60. doi: 10.1089/nat.2023.0038 (PMC10698779; doi:10.1089/nat.2023.0038)
Supplement: Supplemental data [file Suppl_FigS2.pdf]

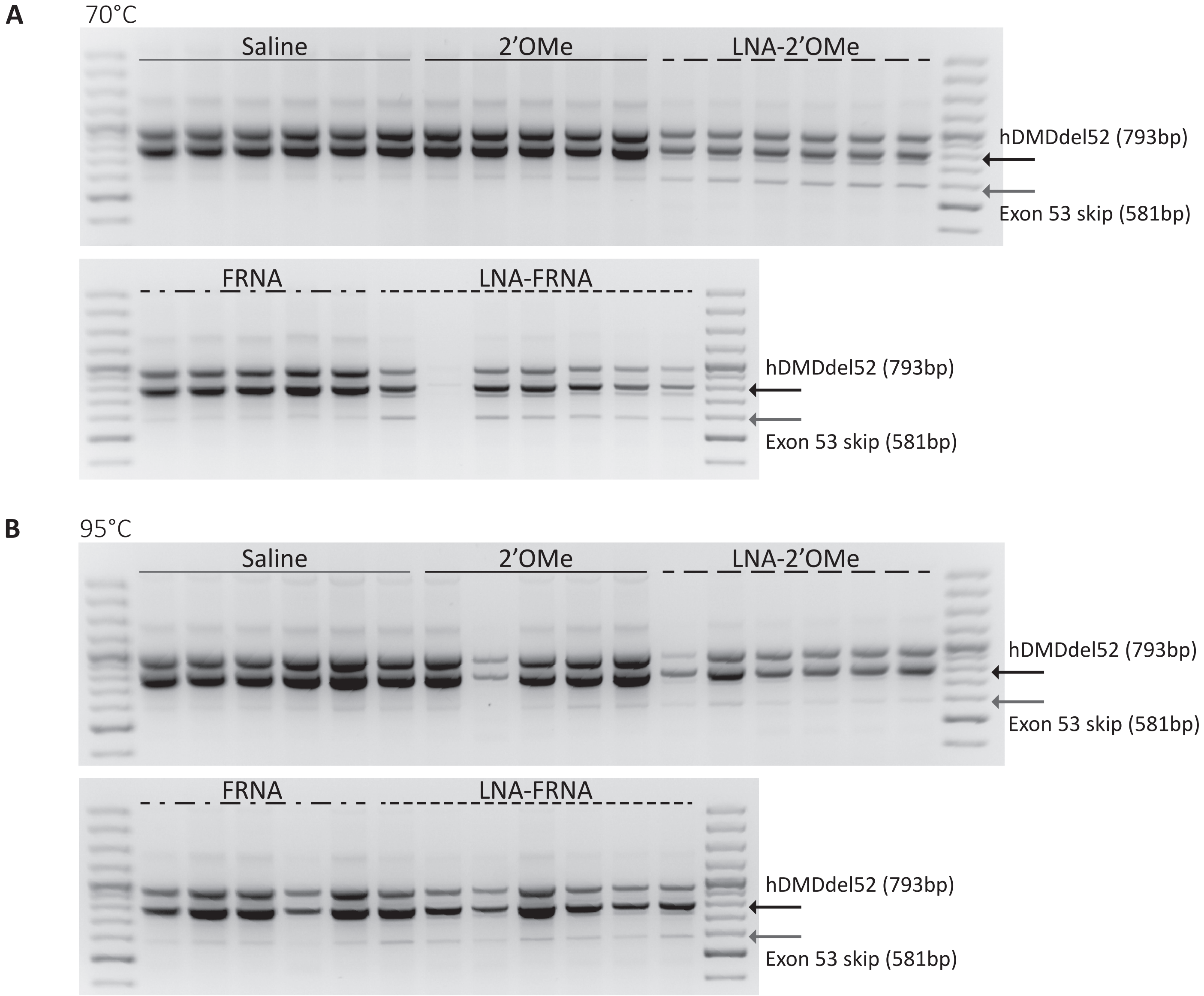

Supplementary Figure 2. Exon 53 skip assessment with a single RT-PCR for the gastrocnemius of hDMDdel52/*mdx* mice after systemic AON treatment. RT-PCR analysis with cDNA synthesized after RNA incubation at **(A)** 70°C or (**B**) 95°C. The hDMDdel52 product contains exons 49-51 and 53-54 (793bp) while the exon 53 skip product contains exons 49-51 and 54 (581bp).
